# Targeting of a Host Protein to Suppress Hepatitis B Virus Replication

**DOI:** 10.1371/journal.pmed.0020237

**Published:** 2005-07-26

Hepatitis B is a serious global public health problem but is preventable with safe and effective vaccines that have been available since 1982. Despite these vaccines, about 2 billion people have been infected with hepatitis B virus (HBV), and more than 350 million have lifelong infections. These chronically infected people are at high risk of death from cirrhosis of the liver and liver cancer, which both kill about 1 million people each year.

Suppression of viral replication in chronic carriers of HBV is an effective approach to controlling disease progression. Current antiviral therapies include lamivudine and alpha-interferon, but long-term resolution of the disease is disappointing because of low seroconversion rates and the development of drug-resistant viral mutants.

In this month's *PLoS Medicine*, Lisa F. P. Ng and colleagues describe the identification of a host factor that has a significant effect on viral replication efficiency. The team began by examining the serum viral load of a group of carriers of hepatitis B in relation to the HBV genome carried. They found a significant association between high serum viral load and a natural sequence variant within the HBV enhancer II regulatory region at position 1752. Upon testing all four possible 1752 variants, the 1752A variant had the highest transcriptional activity.

Further investigation of this enhanced transcriptional activity revealed evidence of possible interaction with host DNA binding proteins. The team found that a protein present in the human host—hnRNPK—could be isolated by direct binding to a viral fragment derived from the HBV variant of these infected patients.

hnRNPK has previously been shown to be involved in several cellular functions—for example, as a regulator of signal transduction and of gene expression. On further examination of the role of hnRNPK in HBV replication, they established that hnRNPK is capable of acting on the full length of HBV, rather than just a partial fragment. They compared four full-length replicative HBV clones, identical except for a single base change at position 1752, that were transfected with two different hnRNPK expression constructs and showed that 1752A was more efficient at promoting replication than the other three variants.

To further show the role of hnRNPK in HBV replication, the team tested the effect of over-expression and down-regulation of the cellular protein. Using siRNA, designed to reduce endogenous hnRNPK, they showed suppression of both hnRNPK mRNA and HBV viral load, whereas a control siRNA had no effect on HBV viral load.

Despite these findings, the mechanism behind hnRNPK on HBV replication needs further exploration, the authors say, concluding that viral replication efficiency was determined by a combination of viral sequence and interaction with specific host proteins. However, they suggest that these results indicate that although drug development of antivirals is an established research avenue, targeting the host is an untapped opportunity.

They describe parallels with anti-EGFR antibody treatment of breast cancer cells, which produced a decrease in cell replication rate and corresponding reduction in hnRNPK expression levels; this result suggested that hnRNPK levels could be modulated by anti-EGFR treatment, thus highlighting new treatment options for altering the HBV viral load in chronic carriers.

The authors conclude that the future of long-term viral clearance will require combination therapy of targeting the virus directly, blocking host support proteins, and using immuno-modulating agents.

